# ShapeMed-Knee: A Dataset and Neural Shape Model Benchmark for Modeling 3D Femurs

**DOI:** 10.1101/2024.05.06.24306965

**Published:** 2024-05-07

**Authors:** Anthony A. Gatti, Louis Blankemeier, Dave Van Veen, Brian Hargreaves, Scott L. Delp, Garry E. Gold, Feliks Kogan, Akshay S. Chaudhari

**Affiliations:** Department of Radiology at Stanford University, Stanford, CA, 94305, USA; Department of Electrical Engineering at Stanford University, Stanford, CA, 94305, USA; Department of Electrical Engineering at Stanford University, Stanford, CA, 94305, USA; Department of Radiology at Stanford University, Stanford, CA, 94305, USA; Department of Bioengineering at Stanford University, Stanford, CA, 94305, USA; Department of Radiology at Stanford University, Stanford, CA, 94305, USA; Department of Radiology at Stanford University, Stanford, CA, 94305, USA; Department of Radiology at Stanford University, Stanford, CA, 94305, USA

**Keywords:** Osteoarthritis, Neural Networks, Magnetic Resonance Imaging, Shape Analysis, Deep Learning

## Abstract

Analyzing anatomic shapes of tissues and organs is pivotal for accurate disease diagnostics and clinical decision-making. One prominent disease that depends on anatomic shape analysis is osteoarthritis, which affects 30 million Americans. To advance osteoarthritis diagnostics and prognostics, we introduce *ShapeMed-Knee*, a 3D shape dataset with 9,376 high-resolution, medical-imaging-based 3D shapes of both femur bone and cartilage. Besides data, ShapeMed-Knee includes two benchmarks for assessing reconstruction accuracy and five clinical prediction tasks that assess the utility of learned shape representations. Leveraging ShapeMed-Knee, we develop and evaluate a novel hybrid explicit-implicit neural shape model which achieves up to 40% better reconstruction accuracy than a statistical shape model and implicit neural shape model. Our hybrid models achieve state-of-the-art performance for preserving cartilage biomarkers; they’re also the first models to successfully predict localized structural features of osteoarthritis, outperforming shape models and convolutional neural networks applied to raw magnetic resonance images and segmentations. The ShapeMed-Knee dataset provides medical evaluations to reconstruct multiple anatomic surfaces and embed meaningful disease-specific information. ShapeMed-Knee reduces barriers to applying 3D modeling in medicine, and our benchmarks highlight that advancements in 3D modeling can enhance the diagnosis and risk stratification for complex diseases. The dataset, code, and benchmarks will be made freely accessible.

## I. Introduction

Osteoarthritis (OA) is the leading cause of pain and disability in developed countries, impacting 30.8 million US adults [1] with an annual US cost of $180 billion [2]. OA affects all tissues in a joint, with emphasis on bone and cartilage. The majority of deep learning research in OA focuses on 2D convolutional neural networks (CNNs) applied to X-rays, 2D and 3D CNNs for segmentation of magnetic resonance images (MRI), and few studies using 3D CNNs for classification of MRIs [3], [4], [5], [6]. OA research largely focuses on X-rays due to the limitations of efficiently processing large 3D image volumes, however, X-rays are a 2D projection of the joint and are thus prone to errors, particularly with repositioning [7].

Characterizing OA relies on medical imaging to discern the shape of anatomic tissues [8]. As OA progresses, osteophytes grow at the edges of cartilage, and cartilage is thinned. Diagnosis of OA is based on these shape features [8]. Beyond OA, shape analysis also serves as the basis for numerous health conditions and diagnoses. For example, shape modeling is crucial for diagnosis and treatment of craniosynostosis, a pediatric condition where skull bones fuse early, causing deformity and potential brain damage [9]. Numerous orthopedic conditions are related to bone shape; both gross shape [10], [11] and nuanced curvatures of joint articulations [12] are important for diagnosing, treating, and preventing disease.

Shape modeling provides an efficient way to analyze 3D anatomic data [13]. However, current shape models, and shape model research has limitations. Widely adopted statistical shape models (SSMs) require anatomic point matching, which is not guaranteed and, in disease, may not be possible. For example, osteophytes that form in OA are not present in healthy bones, and thus no true matching points exist. Once matching points are obtained, SSMs are typically fit using linear statistical representations, namely principal components analysis (PCA); shape features of disease are unlikely to be purely linear in nature. Applications of SSMs in medicine are typically used to identify gross features or predict disease in general [14], [15]; accurate quantification of specific, localized, biomarkers of disease are required for clinical applications. To advance shape analysis in medicine, we require benchmarks that assess clinically relevant reconstruction metrics, and whether a model can localize relevant disease features.

**Fig. 1.**
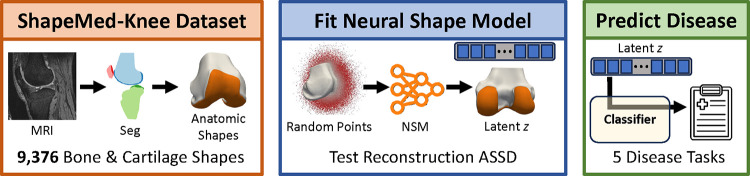
The ShapeMed-Knee dataset was created by segmenting and meshing 9,376 knee MRIs (orange box). We fit three shape models, two neural shape models (NSM) and one statistical shape model (SSM) to the ShapeMed-Knee training data and evaluated reconstruction tasks, including average symmetric surface distance (ASSD) (blue box). To test latent vectors z learned by the shape models, we train and evaluate classifiers for five clinical tasks (green box).

With our overarching objective to enable the advancement of medical domain-specific 3D modeling, we provide the following contributions (Fig. 1):

- We introduce ***ShapeMed-Knee*:** a 3D anatomic dataset with 9,376 shapes, each including two interrelated objects (femur bone and cartilage). We publicly share segmentation masks, and 3D shapes.
- We define seven medically relevant benchmark tasks with our ShapeMed-Knee dataset: surface reconstruction, cartilage biomarker calculation from reconstructions, disease diagnosis, localized disease staging, and future surgical event prediction.
- We develop hybrid explicit-implicit neural shape models (NSM) that outperform both SSMs and implicit NSMs for bone and cartilage reconstruction (7–20% lower average symmetric surface distance).
- We demonstrate that our hybrid NSM outperforms an SSM, implicit NSM, and CNN in disease staging, disease diagnosis, and localization of specific features of disease.
- We show that interpolation in NSM latent space produces interpretable smooth interpolation of physical shape, clinical shape features, and clinical predictions.
- We demonstrate precise control over localized disease features by interpolating latent space along classifier-fitted vectors, enabling targeted manipulations of disease characteristics.
- We publicly share our NSM model and the code used for training and inference. A tutorial on how to download and used the data is provided at https://github.com/gattia/shapemedknee.

## II. Related Work

Neural representations have advanced computer graphics [16]. ShapeNet data has been central to the advancement of generative 3D shape models [17]. The recently proposed MedShapeNet is similar to ShapeNet, but includes 3D anatomic shapes with multiple inter-related tissues [18]. However, there still exists a gap in 3D anatomic models with curated disease-specific reconstruction metrics and clinical tasks; these data are needed to enable focused research that advances methods for quantifying anatomic shapes and understanding how these shapes influence health and disease.

### A. Generative Implicit Neural Representations

DeepSDF was the first reported use of a generative implicit neural representation [19]. DeepSDF uses a multilayer perceptron (MLP) to generate shapes conditioned on a latent vector z. DeepSDF enables shape compression, interpolation, and completion from partial observations. Numerous DeepSDF advances have been proposed. Curriculum DeepSDF using curriculum learning [20]. Modulated Periodic Activations combine two MLPs as a means of leveraging periodic (sinusoidal) activations, which outperformed rectified linear unit (ReLU) MLPs for single object reconstruction [21], [22].

To improve reconstruction of large scenes or fine details, instead of a single global *z,* a spatially localized z is input into the MLP [23], [24]. Hybrid explicit-implicit formulations generate localized z by leveraging the expressivity of CNNs [24], [25], [26], [27]. Both generative adversarial network and variational autoencoder (VAE) frameworks have been used in these hybrid explicit-implicit models [25], [26].

### B. Shape Modeling

Shape modeling has many important applications for biomedical data. In just the OA community, shape models have been used for automated segmentation [28], [29], disease prediction and staging [15], [30], [31], and generating synthetic data for physics-based simulations [12], [32]. Shape models have advanced understanding and treatment of conditions related to the heart, brain, skull, and bones, to name a few [33], [9], [34], [10], [11]. Improved shape modeling can benefit all of these areas, providing tangible benefits in understanding disease and improving patient health.

### C. Statistical Shape Models

Conventional SSMs use PCA to learn shape features. The main challenge with PCA-based SSMs for anatomical objects is the need for matching points at the same anatomical location on each object. Correspondence is typically obtained via nonrigid image registration of signed distance fields [28], or non-rigid point cloud registration [14], [12], [35]. To improve anatomic correspondence, registration features beyond XYZ coordinates, such as spectral coordinates or curvatures have been included [14], [36]. Registration is prone to failure in abnormal or diseased areas, which are typically the most important.

### D. Neural Shape Models

We refer to generative shape models in the medical domain as NSMs. There are only a handful of NSM applications. Amiranashvili et al. fit an occupancy NSM to anisotropic bone data showing occupancy-based methods can be trained and applied to undersampled anisotropic data. However, the occupancy NSMs still exhibit relatively large reconstruction errors (average symmetric surface distance (ASSD): 0.25–0.48mm) [37]. Jensen et al. fit a NSM by deforming points on a sphere using point-specific latent vectors. During training, a single latent vector was used for all points, while during inference, latents vary over the surface to increase expressivity. They showed better reconstruction than DeepSDF and improved segmentation results [38]. Ludke et al. used a neural flow deformer to fit a NSM by deforming coordinates from a template shape to the target, outperforming a conventional SSM in terms of surface reconstruction and simple OA classification [39].

Biomedical research demonstrates that implicit neural representations applied as NSMs improve anatomical reconstructions and image segmentation results and can encode basic clinical information. However, existing work represents only a single tissue at a time, uses relatively small samples of data (41–354 examples), and primarily focuses on surface reconstruction results rather than the quality of learned representations. Finally, biomedical approaches are challenging to compare as they use different datasets and downstream prediction tasks.

## III. Dataset & Evaluation

Data from this study is derived from the Osteoarthritis Initiative (OAI), a multi-center, longitudinal observational study of 4,796 men and women (45–79 years of age) with the goal of developing biomarkers of OA. The OAI collected patient clinical data, X-rays, and MRIs annually for 9 years. Important for the prediction tasks in this study, teams of expert radiologists were contracted to label acquired images for OA diagnosis, as well as standardized features of OA disease. We derive our dataset from the MR imaging data collected at the baseline time point and the radiologist evaluations from the baseline and all follow-up time points.

used stratified random sampling to split the OAI baseline data into train/validation/test sets at the subject level, as right and left joints can be highly correlated and provide a form of data leakage. Splits were stratified over sex and clinical prediction tasks (III) to ensure disease states and outcomes were equally represented. Due to the iterative and time-consuming nature of fitting the NSM during inference, a small validation set was used in this study (train: 67.5%, 3,233 people and 6,325 knees; validation: 2.5%, 74 people and 141 knees; test: 30.0%, 1,481 people and 2,910 knees). Tab. I contains an overview of the included data.

### A. ShapeMed-Knee Dataset Creation

#### 1) Segmentations & Surfaces:

We extracted 9,376 Double Echo in Steady State (DESS) knee MRIs from the baseline visit of participants in the OAI [40]. We segmented DESS MRIs automatically using a multi-stage CNN framework; this approach was validated on the OAI dataset, achieving Dice similarity coefficients of 0.99 and 0.91 for femoral bone and cartilage and low ASSD (0.08–0.15mm) [41]. This performance is equivalent to the best-reported cartilage segmentations [6], [29], and is the same as expert-human level in terms of cartilage sensitivity to change [42]. All left knee MRI segmentations were flipped to create right knees and remove variance due to anatomical side. Three-dimensional surfaces were then generated from each femur bone and cartilage segmentation mask using previously established methods [35]; code to create surface meshes is shared for reproducibility.

##### a) Cartilage Thickness Biomarker:

**Fig. 2.**
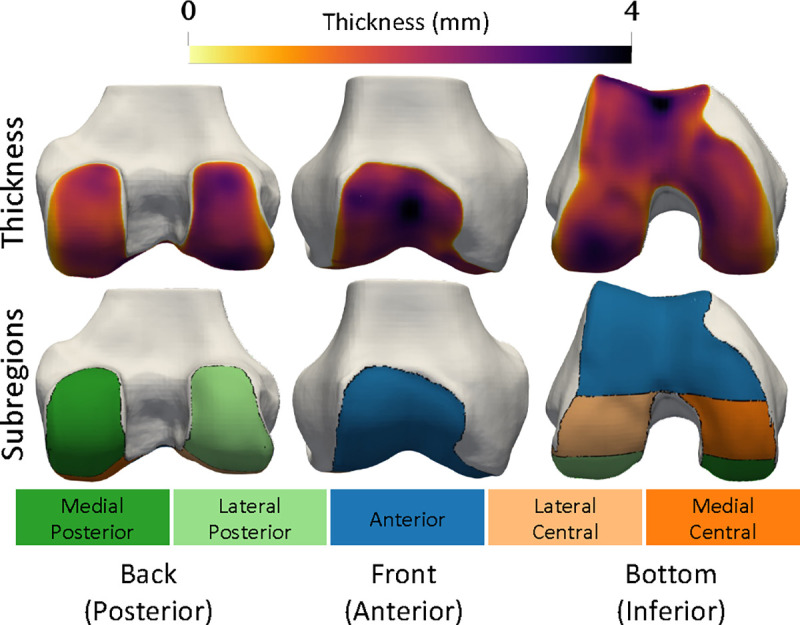
Cartilage thickness (top row) and subregions (bottom row) are displayed on the bone surfaces. Blue is anterior (front), orange is central (middle in front/back axis), green is posterior (back). Dark colors denote medial i.e. the inside of the knee, while light colors denote lateral i.e. the outside.

Mean cartilage thickness in pre-defined anatomic regions is a common biomarker for clinical trials and experimental studies [43], [44]. It is critical that NSM-reconstructed surfaces preserve these biomarkers relative to reference surfaces [45]. We calculated cartilage biomarkers with the following processing steps: i) divide cartilage segmentations into subregions, ii) compute cartilage thickness for each vertex over the bone surface, iii) assign each bone-vertex to one of the subregions. Cartilage biomarker calculations used open-source code [46] used in previous investigations [35], [47]. From these data, we computed five cartilage thickness biomarkers as the mean thickness for all bone mesh vertices in each of five established cartilage subregions (trochlea, medial central, lateral central, medial posterior, lateral posterior) [48]. Visualization of cartilage thickness, subregions, and a general orientation to the data are presented in Fig. 2.

##### b) Bone Surface Registration:

All femur bones were co-registered to have matching points to create a traditional SSM (Sec. IV) as a baseline model; original full resolution meshes (~220,000 points) were used for the NSMs. First, to reduce the computational complexity of the registration, each bone mesh was downsampled to 20,000 vertices [49], [50]. Next, an average femur shape, determined from 281 knees in a prior study [51], was used as the template and nonrigidly registered to every other bone in the dataset using spectral correspondence-based registration [52], [36] that has been used in multiple knee OA studies [35], [14]. Cartilage thickness and subregions were re-calculated for the registered meshes as described in the previous section Sec. III-C.2. The resulting registered meshes included matching points and cartilage thicknesses for 9,376 femur bones.

##### c) Mesh Quality Control.:

To ensure high-quality meshes in the dataset, we generated static images of every bone mesh from 4 orthogonal planes (top, bottom, front, back) using pyVista [53] and an imaging researcher with 10 years of experience with bone analysis manually reviewed every image. From this analysis, we identified 57 meshes (0.6%) with large errors primarily due to physiologically-plausible holes at the sites of anterior cruciate ligament reconstruction. These 57 meshes were removed from the dataset. An additional 9 meshes had moderate errors, and 30 meshes had small potential errors; these meshes were retained in the dataset. IDs for moderate and small error meshes, and quality control images for all knees are provided for dataset users to use their custom exclusion criteria.

### B. Prediction Tasks

OA is a whole joint disease that affects multiple tissues, with an emphasis on the cartilage and bone. We developed five prediction tasks which test a model’s ability to understand shape complexity relevant to current bone and cartilage health as well as future disease progression.

OA is commonly diagnosed using X-rays graded using the Kellgren-Lawrence (KL) system [8]. The KL system assigns knees a grade between 0–4 (0 = no OA, 1 = doubtful OA, 2 = mild OA, 3 = moderate OA, 4 = severe OA). Diagnosis with OA is defined as KL≥2. Beyond diagnosis, KL grading is used in research and clinical trials to “stage” the severity of OA in the whole joint (all tissues/bones) beyond binary classification. Therefore, our first two tasks are:

1. **General OA staging** by predicting KL grade (0–4)
2. **Binary OA diagnosis** (KL≥2)
  While KL grading provides a whole-joint OA measure, it is a coarse measurement based on 2D X-rays and does not provide fine-grained, location-specific information in 3D. Therefore, it cannot be used to identify where and what tissues are involved in a person’s disease. The MRI Osteoarthritis Knee Score (MOAKS) measures multiple features of OA that are localized to different regions of the joint [54]. Our third task involves predicting three MOAKS scores (one bone and two cartilage features) in six distinct regions of the femur. MOAKS scoring provides clinically important information and can simultaneously serve as a test of how well a model can spatially localize fine-grained OA features. Task three is:
3. Advanced localized OA staging by predicting three MOAKS scores (Score 1: Osteophytes, Score 2: Cartilage Thinning, Score 3: Cartilage Hole) in 6 femoral regions divided across the anterior, central, and posterior regions in the medial and lateral condyles.

The three MOAKS scores were defined as follows:

- ***Score 1 Osteophytes*:** Osteophytes are abnormal bone growths (bone spurs) that occur at the edges of the cartilage and are a hallmark sign of OA. The MOAKS osteophyte score includes 4 levels (0: None, 1: small, 2: medium, 3: large). Due to a low prevalence of grade 3 scores (< 5%), we binned MOAKS osteophyte score into 3 levels (0–2) where level 2 includes original scores of 2/3.
- ***Score 2 Cartilage Thinning*:** A key sign of OA is cartilage thinning. The MOAKS cartilage thinning score categorizes the % of a region with any cartilage thinning into 4 categories. Given a class imbalance amongst the four categories, we binarize this score as individuals with < 10% thinning (grades 0/1) and > 10% thinning (grades 2/3). This approach is used in prior OA studies [55].
- ***Score 3 Cartilage Hole*:** The final score quantifies the % of a region that has a full thickness defect (a hole) in the cartilage into the same 4 levels (0–4) as cartilage thinning. Cartilage holes rarely occur (6–16%), thus we binarized this score into no hole (grade 0) and any hole (≥ 1).

The final two tasks were created to test whether a model can predict future OA diagnosis (within 4 years) in currently healthy subjects, and whether a medical event (knee replacement) has occurred (within 9 years). Future OA diagnosis and knee replacement prediction are common tasks performed in the OA literature, are challenging, and would provide valuable information to identify which patients should be treated earlier. MRI-based SSMs of bone shape, and CNN’s applied to X-ray data have previously been used to predict these outcomes [15], [30], [56], [57].

- **Predict future disease** (**OA**) within 4 years.
- **Predict future knee replacement** within 9 years.

### C. Evaluations

#### 1) Surface Reconstruction:

We evaluate surface reconstruction errors separately for the bone and cartilage surfaces using ASSD. We test ASSD on the whole test set and separately for the 5 KL grades to assess whether reconstruction errors depend on disease state.

#### 2) Cartilage Thickness Biomarker:

To evaluate whether reconstructed bone and cartilage surfaces preserve important cartilage biomarkers, we analyze the five cartilage subregions on the whole test set and on each of the 5 KL grades in the test set. Between the mean thickness of the original and reconstructed surfaces we compute 1) the root mean squared error (RMSE ↓) to determine absolute errors and 2) the standard deviation of the difference (*SDD* ↓) as a measure of consistency that removes the effect of systematic bias.

#### 3) Prediction Tasks:

- ***OA Staging.*** OA staging is quantified using the KL grade, a semi-quantitative multi-class measure of OA with variation between raters. As such, relative agreement is commonly used to assess KL predictions and inter-rater agreement. We use accuracy and quadratically-weighted Cohens Kappa, as done previously [58], [59], [60].
- ***OA Diagnosis.*** As OA diagnosis is a binary prediction task with relatively well-balanced groups, we compute the common metrics of area under the receiver operating characteristic curve (AUROC) and accuracy.
- ***Advanced OA Staging (MOAKS).*** We assess three MOAKS scores (measuring osteophytes, cartilage thinning, cartilage holes) separately for six regions of interest. Score 1 (osteophytes) includes three classes, and thus we compute quadratically weighted Kappa and accuracy. Since both Score 2 (cartilage thinning) and Score 3 (cartilage hole) are binary tasks with large class imbalance, we compute F1 score and the area under the precision-recall curve (AUPRC).
- ***Future disease (OA).*** The incidence of OA in the four years following baseline was relatively rare, occurring in only 9% of subjects. Therefore, we compute the F1 score and AUPRC.
- ***Future knee replacement surgery.*** The incidence of knee replacement in the 9 year follow-up was rare (5%). Therefore, we compute the F1 score and AUPRC.

## IV. Benchmark Models

We compared multiple types of shape models and CNNs on our tasks. We compare an SSM, implicit NSM, and our hybrid explicit-implicit NSM for reconstruction tasks. In addition to these models, for the prediction tasks, we also compare 3D CNNs applied to raw image data and to bone/cartilage segmentations. The models are described in the following.

### A. Neural Shape Models

DeepSDF-based NSMs train a decoder to take as input a latent vector z and coordinate x and predict the signed distance s of x. NSMs typically use an autodecoder framework where z is learned by jointly optimizing a dictionary of latents along with the network weights to predict *s* while using regularization so z matches a multivariate Gaussian distribution. Both NSMs used in this study were trained using the same framework, including point sampling, training hyperparameters, and reconstruction strategy.

#### a) Point Sampling:

Before training, an arbitrary mesh was chosen as the reference. Every other bone mesh was registered to the reference using a similarity transform (rigid + scale); the transform was applied to the coinciding cartilage surface. Next, bone and cartilage meshes were centred using the mean of the bone points and were normalized using maximum radial distance so both tissues lie within a unit sphere. Then, separately for the bone and cartilage surfaces, 500,000 points were sampled. Ninety percent of points were randomly sampled by first sampling positions on the surface using blue noise to produce uniform random samples. Then, sampled surface points were perturbed by adding zero mean Gaussian noise: 45% σ=0.016 ;45% σ=0.05. The remaining 10% of points were uniformly sampled over the unit cube. Finally, *s* from both meshes was calculated for every sampled point.

#### b) Training:

Prior to training, each bone/cartilage pair was assigned a random $z∼𝒩\left(0,0.01^{2}\right)$. During training, for each subject (k ) and surface type (j: bone/cartilage), 17,000 points ($X_{jk}$ ) were randomly sampled with equal numbers of points inside (−) and outside (+) the surface. Eqn. (1) was optimized to minimize the error in predicted s and to regularize the latent z. The loss comprises a reconstruction and latent regularization term. The reconstruction term penalizes hard samples (predicted wrong sign) as shown in Eqn (2) and includes a weighted ${ℒ}_{1}$ where λ(0−1) controls the weighting on hard samples with λ=0 being equivalent to regular ${ℒ}_{1}$ and higher values provide greater penalty [20]. λ was exponentially increased from 0 to 0.2 over the first 1800 epochs. A latent regularization loss independently penalized each z component with σ=100 to promote diagonal covariance. Latents and network weights $f_{\theta }$ were jointly optimized using the AdamW optimizer with weight decay of 1e-4 [61].


(1)

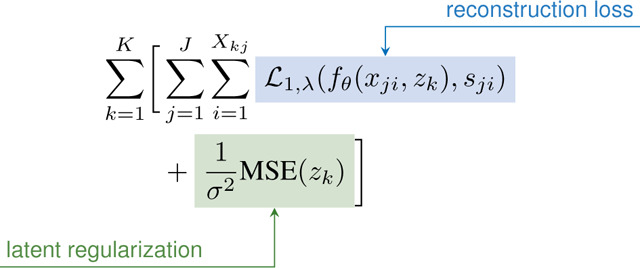




(2)

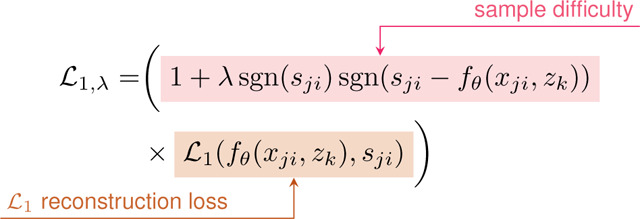



#### c) Reconstruction:

To reconstruct surfaces and create shape-specific latents, the NSM weights were frozen and the NSM was fit to the new surfaces. Specifically, the bone to be reconstructed was similarity registered to the mean bone shape of the NSM (zero-vector) and the bone/cartilage surfaces were scaled to be within a unit sphere. Then, a randomly initialized latent $z∼𝒩\left(0,0.01^{2}\right)$ was optimized for 2,000 epochs to reconstruct the surfaces using an ${ℒ}_{1}$ loss between the network predicted signed distance *s* and the actual *s* of 20,000 randomly sampled surface points (*s* = 0) using the Adam optimizer. The lr was decayed by a factor of 0.9 every 20 epochs, and early stopping was implemented with a patience of 50 epochs.

#### d) Hybrid Explicit Implicit NSM:

The hybrid NSM is based on triplanar architectures [25], [26] as outlined in Fig. 4. A global latent z of a length of 512 is processed via a fully connected layer, resulting in a 2048-length vector. This vector is then reshaped to be 2 × 2 × 512 before being input into a CNN decoder. The CNN decoder had 5 2D transpose convolution layers, with stride 2 and 512 channels as outputs at each layer. The final output layer of the CNN was sized 64 × 64 × 384; the 384 features maps were split into 128 features per orthogonal plane. Sampled points $x\in R^{3}$ are projected onto the three orthogonal planes, and a length 128 z was obtained per feature plane via bilinear interpolation. Plane features were combined via summation, yielding a length 128 local z. The local z and the sampled x position were concatenated and input into the implicit 3-layer MLP with width 512, *ReLU* activations, and a length two output (one for each tissue) with a tanh activation.

#### e) Implicit NSM Network:

The implicit decoder was an 8-layer MLP of width 512, with a skip connection of the inputs (x and z) to layer 4 and ReLU activations throughout. The output was sized two and used the tanh activation.

### B. Statistical Shape Model

The SSM was fit using [46], the same as described in previous investigations [35], [14]. SSM-based reconstruction does not provide explicit cartilage surfaces but instead computes thicknesses at each bone vertex, therefore ASSD was not evaluated for SSM cartilage.

### C. Convolutional Neural Network

We trained two DenseNet121 models as implemented in the MONAI package [62]. One network was trained with an input of the raw DESS MRI data and the other an input of the bone/cartilage segmentations. For both variants, the 3D volumes used for input were downsampled from the original volumes (384 × 384 × 160) to be sized 384 × 384 × 80, using bilinear interpolation. This approach preserved full-resolution data in-plane, while reducing slice thickness to 1.4mm, which is sufficient for clinical trials including quantitative cartilage analyses [44]. CNNs were trained with the AdamW optimizer, an initial learning rate of 10^−5^ exponential decay with gamme=0.8 and weight decay=0. Training was performed with a single Nvidia A6000 GPU.

## V. Experiments

### A. Reconstructions

Reconstruction evaluations are provided for the SSM, implicit NSM, and hybrid NSM. No reconstruction results are provided for the CNN because it is not generative.

#### a) Dataset Size:

To determine data efficiency, we trained each shape model using 4 training set sizes: 50, 200, 1,000, 6,325. NSMs were trained for 2,000 epochs (Tab. IV). SSMs were tested using progressively more principal components (Tab. IV). These analyses identified that: a) The hybrid NSM performed best for ASSD and both cartilage biomarker measures across dataset sizes, b) Increasing dataset size up to 6,325 increased reconstruction performance for all models, and c) Increasing the number of PCs used in SSM reconstruction did not overfit up to 1,298 PCs (99% explained variance)

**Fig. 3.**
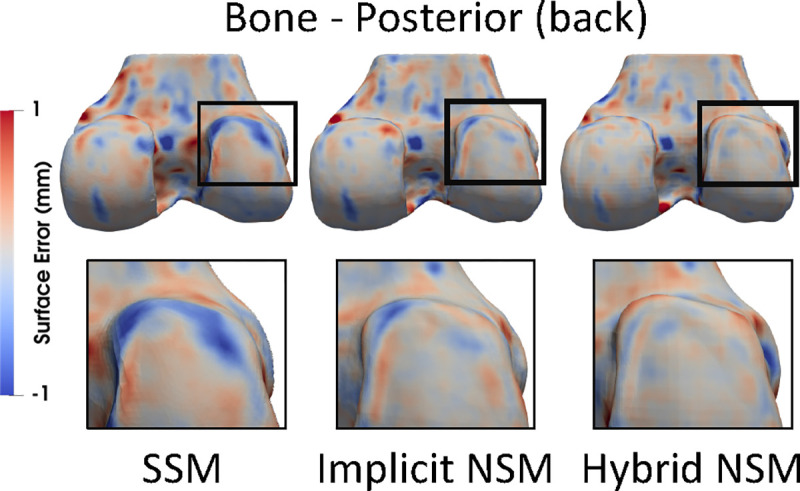
Reconstructed bone and cartilage surfaces colored by reconstruction error. Blue indicates the reconstruction was inside of the reference, and red indicates the reconstruction was outside. Zoomed regions highlight an area of disease (osteophyte on the posterior lateral femur) that was not captured by the SSM (blue), had smaller error for the implicit NSM, and had the least error for the hybrid NSM.

**Fig. 4.**
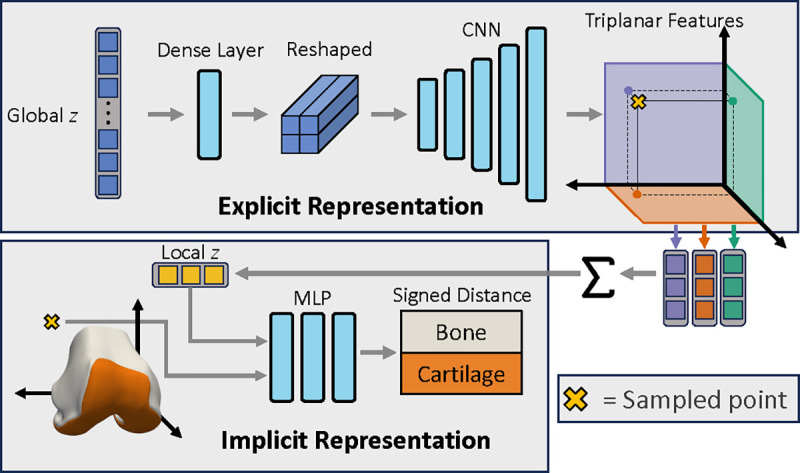
Overview of network architecture. A global latent z controls the overall generated shape. The global z is passed through a dense layer, reshaped and then fed through a 5-layer CNN to produce 64 × 64 2D output with 384 feature maps. The 384 feature maps are split into 3 to produce one set of 64 × 64 × 128 feature maps per orthogonal plane. To determine the signed distance of a particular point (⊗) that point is projected onto each feature map plane, and the corresponding feature vector is extracted using bilinear interpolation. These plane-specific feature maps are summed, yielding the local **z**. The local z is a coordinate-specific latent vector that controls the signed distance prediction. The local z along with the XYZ coordinates of point ⊗ are passed to a three-layer multilayer perceptron which outputs the signed distance of the two surfaces (bone and cartilage).

**Fig. 5.**
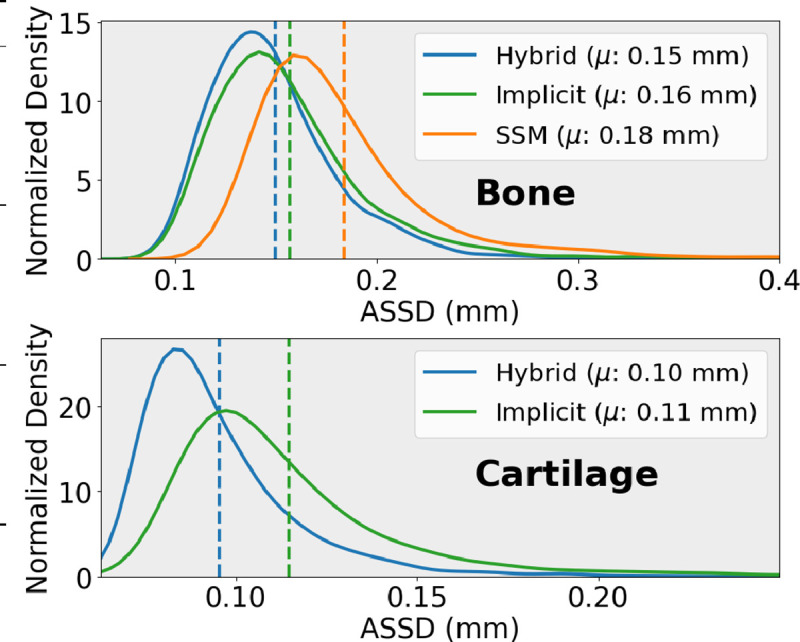
Probability density functions of the bone and cartilage average symmetric surface distances (ASSD). Distribution tails were truncated for visualization purposes.

The hybrid NSM better reconstructed areas of OA disease (Fig. 3). Fig. 5 distributions of ASSDs in the test set demonstrate that the hybrid NSM had better ASSD for bone (6–17%) and cartilage (9%). Tab. II shows that when assessed for all data, as well as by KL grade, the hybrid NSM had the lowest errors for reconstruction and cartilage biomarkers. Better SDD compared to RMSE indicates that all models had a small bias compared to the reference standard (Tab. II).

#### b) Latent Size:

We tested the effect of doubling latent size on ASSD errors for the hybrid and implicit NSM models. Reconstruction accuracy improved as latent size increased, with the hybrid NSM ASSD dropping 26% and 22% for bone and cartilage, respectively (Tab. IV).

### B. Classification / Staging

An MLP was trained to predict each clinical evaluation task using each model’s encoded z as input. Hyperparameters were determined via a grid search over depth (2,3), width (64–256), dropout (0.2, 0.4), learning rate (10^−3^ to 10^−5^), and batchsize (64–512). We also trained two 3D CNNs for clinical prediction tasks Sec. IV-C. Loss functions for CNNs and MLPs included binary cross entropy (OA, MOAKS cartilage thinning and hole, future OA and knee replacement) and consistent rank logits ordinal regression (KL, MOAKS osteophytes) [63].

#### a) OA Staging & Diagnosis:

For predicting KL, the resulting κ of the trained models was 0.69–0.79, with the hybrid NSM having the best performance and the implicit NSM having the worst Tab. III. All models performed comparably to inter-radiologist agreement (0.66–0.89)[59], [64], [65], [60]. Prior X-ray based DL methods performed slightly better (0.83–0.88) [58], [60].

When directly diagnosing OA, the hybrid NSM performed best (AUROC: 0.92) and the implicit NSM performed worst (Tab. III), similar to the KL task. Interestingly, the CNN applied to the segmentation and the image performed the same, indicating the raw MRI provides no additional information. Accuracy was slightly lower (0.81–0.83) than DL-based X-ray OA grading (0.87–0.90) [66], [60], likely because X-rays are the original data used to grade KL. However, the 2D X-ray projection of the joint is prone to positioning errors [7] and thus it is possible that 3D analyses are closer to the ground truth physiologic (not image-based) grading. Our CNN predictions were comparable to a previous CNN applied to MRI data for predicting OA [67].

#### b) Advanced OA staging:

The hybrid NSM performed best for all three MOAKS tasks when averaged over the regions (Tab. III). These results indicate that the latent z fit by the NSM more meaningfully represented both the location and the size of OA features. Not only is this important for OA, but it demonstrates novel capacities of NSMs that are not commonly tested; the ShapeMed-Knee dataset provides a unique method of testing these capacities using real-world data.

The CNN models performed poorly in identifying cartilage holes (F1: 0.00–0.03) and were no better than chance for the MOAKS osteophyte tasks (κ0−0.04) Tab. III. Prior DL work uses MOAKS to determine severity of cartilage damage [55]. Other work predicts other features of MOAKS, bone bruises [68] or inflammation [69]. This is the first quantification of MOAKS osteophyte and cartilage health, demonstrating that NSMs encode this important information that is currently prohibitive to obtain clinically, and costly for research and clinical trials.

#### c) Future OA & knee replacement prediction:

All models performed poorly on future event prediction tasks (Tab. III), despite prior SSM bone shape work showing links between current shape and future disease [15], [31]. However, these prior studies used odds ratios to determine if certain shapes are more likely to get worse, and did not always use a test set [15]. The best-performing future OA diagnosis was by the raw image-based CNN (AUPRC: 0.20, F1: 0.26); it is possible non-shape-related features such as bone bruises or joint inflammation boosted CNN image performance [70].

### C. Interpretability

**Fig. 6.**
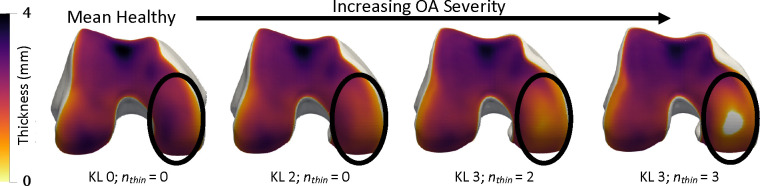
Interpolation in hybrid NSM shape space along the mean healthy to the mean severe OA axis. Smooth progression of cartilage thinning occurs on the medial central femur (circled) with a hole (grey) occurring at the end. Each bone is annotated with disease stage classifications determined by logistic regressions, KL grade, and the number of regions with cartilage thinning ($n_{\text{thin}}$).

One of the powers of shape models is that they are fit in a self-supervised fashion, and are generative. To show the utility of this, we trained a logistic regression classifier on hybrid NSM z for each prediction task. Results in Tab. III show that the simple classifier is one of the best for disease staging. We tested latent interpolation smoothness by assessing the effect of interpolation on reconstructions and disease prediction. Using the hybrid NSM we interpolated z from the mean healthy (KL 0) to the mean severe OA (KL 4) shapes in the test set, generated synthetic surfaces, and applied the logistic classifiers on each z to determine KL and MOAKS cartilage thinning grades Fig. 6. Shape space interpolation generated smooth physical interpolations and predicted smooth transitions of disease states Fig. 6. This general-purpose representation is powerful because application to other image modalities only requires a segmentation mask, whereas CNN-based approaches would require re-training on entirely new datasets. Furthermore, interpolation could be used to track individual patient disease trajectories over time, opening the door to novel ways of understanding disease.

**Fig. 7.**
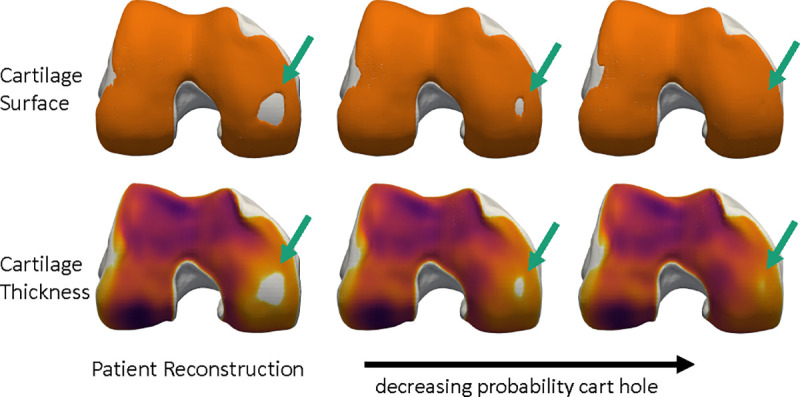
Interpretation of the logistic regression-based MRI Osteoarthritis Knee Score (MOAKS) medial cartilage hole classifier. The top and bottom rows are of the same bone, showing the solid cartilage surface (top) vs. the thickness map (bottom). The left column is the NSM reconstruction of a patient with a medial cartilage hole. The other two columns are synthetic bone and cartilage surfaces generated by interpolating the patient-fitted latent z along a vector defined by the logistic regression coefficients. The synthetic bones progressively close the cartilage hole, while generally leaving the other bone and cartilage regions the same. Specific control of anatomical features indicates that these features can be monitored longitudinally and that synthetic alternatives to patient anatomy can be generated for *in silico* simulations.

The generative nature of the NSM enables further validation that classifiers applied to the latent z are capturing features of interest. Fig. 7 takes the latent z fitted to a patient, and interpolates it along the vector defined by a logistic regression classifier that predicts medial cartilage holes. Simple linear interpolation along the fitted vector precisely controls the size of the cartilage hole on the medial side. This visualization improves confidence in the fitted model, but may also enable entirely new applications. For example, it is possible to precisely add and remove specific, localized, features of disease and therefore to generate synthetic versions of a patient’s anatomy. These synthetic digital twins can be used for *in silico* simulations to determine the effects of specific disease features on tissue biomechanics [12], or to inform surgical planning such as cartilage repair [71], [72]. Importantly, this example uses simple linear interpolation; future work can leverage latent diffusion models [73] to advance this capacity.

## VI. Conclusion

We contribute a hybrid explicit-implicit NSM which demonstrates state-of-the-art performance for anatomic reconstruction, and clinical outcome prediction. Model training and evaluation were enabled by our new ShapeMed-Knee dataset. All shape models were capable of simple OA staging. Hybrid NSMs uniquely quantified the location and size of OA features. While hybrid NSMs provide current state-of-the-art bone and cartilage reconstruction, further advances applied to our ShapeMed-Knee dataset have the potential to improve results and, in turn, our understanding of OA. We encourage the community to leverage ShapeMed-Knee data and benchmarks to tackle the unique challenges presented by modeling multiple anatomic surfaces and encoding meaningful disease-specific information.

## Figures and Tables

**TABLE I:** Data Extent at the knee level for evaluations.

Task	Train	Val	Test	Total
	
Subjects	3,233	74	1,481	4,788
Recon	6,325	141	2,910	9,376
KL / OA	5,919	128	2,371	8418
MOAKS (O)	403	0	194	597
MOAKS (C)	1,414	0	688	2,102
Future OA	3,385	76	1,534	4,995
Future KR	6,325	141	2,910	9,376

*Subjects* is the number of individuals in each datasplit. *Recon* is the number of 3D models. *Current Osteoarthritis (OA)* grade quantifies disease severity. MRI Osteoarthritis Knee Score (*MOAKS*) quantifies Osteophyte (O) and Cartilage (C) outcomes. *Future OA* quantifies healthy to OA progression in 4 years *and Future knee replacement (KR)* quantifies surgery in 9 years.

**TABLE II:** Summary of reconstruction performance for each model (SSM, implicit NSM, hybrid NSM) across the whole test dataset (All) and each KL grade (0–4). Metrics include surface reconstruction errors (ASSD) and cartilage biomarker outcomes (RMSE, SDD) averaged over five regions.

Metric	Group	SSM	Implicit	Hybrid
		
ASSD ↓, Bone/Cart (mm)	KL 0	.16	**.14** / .10	**.14 / .08**
	KL 1	.17	.15 / .10	**.14 / .09**
	KL 2	.19	.17 / .12	**.16 / .10**
	KL 3	.21	.18 / .13	**.17 / .11**
	KL 4	.32	.25 / .20	**.22 / .15**
	All	.18	.16 / .11	**.15 / .10**

Average RMSE ↓ SDD↓	KL 0	.04 /.03	.05 / .04	**.03 / .02**
	KL 1	.04 / .04	.04 / **.03**	**.03 / .03**
	KL 2	.05 /.04	**.04** / .04	**.04 / .03**
	KL 3	.06 /.05	.05 / .04	**.04 / .03**
	KL 4	.10 / .08	.14 / .14	**.05 / .04**
	All	.05 /.04	.06 / .05	**.04 / .03**

**TABLE III:** Performance on the prediction tasks using metrics described in Sec. III-C.3. Hybrid NSMs consistently exhibit the best performance.

Task	Metric	CNN Seg	CNN Image	Method SSM	Implicit	Hybrid	Hybrid+LR
		
KL	*κ* / Acc	.75 / .59	.78 / .59	.78 / .59	.69 / .54	**.79** / .59	.72 / **.60**
OA	AUROC / Acc	.90 / **.84**	.90 / .81	.91 / .83	.88 / .80	**.92** / .83	**.92** / .81
MOAKS Osteo	*κ* / Acc	.00 / .49	.04 / .50	.16 / .50	.35 / .54	**.53 / .63**	.46 / .60
MOAKS Cart Thin	AUPRC / F1	.51 / .23	.50 / .31	.63 / .51	.53 / .50	.74 / **.66**	**.75** / .63
MOAKS Cart Hole	AUPRC / F1	.32 / .00	.31 / .03	.41 / .16	.44 / .40	**.57 / .55**	.56 / .33
Future OA	AUPRC / F1	.10 / .18	**.20 / .26**	.10 / .19	.15 / **.23**	.12 / .19	.14 / .18
Future KR	AUPRC / F1	.07 / .13	.29 / **.34**	.27 / .33	.24 / .32	**.33** / .28	.18 / .27

*κ*: quadratically-weighted kappa; Acc: Accuracy; AUROC: area under the receiver operating characteristic curve; AUPRC: area under the precision recall curve; OA: osteoarthritis; KR: knee replacement; LR: logistic regression. All shape models used an MLP, except for hybrid+LR which used LR.

**TABLE IV:** Validation set (n=141) reconstruction performance for multiple dataset and latent sizes.

Model	Latent size	Dataset size	ASSD Bone	ASSD Cartilage
			
Hybrid NSM	512	50	0.27	0.19
	512	200	0.20	0.14
	512	1,000	0.17	0.11
	512	6,325	0.15	0.09
	1,024	6,325	**0.11**	**0.07**

Implicit NSM	512	50	0.37	0.40
	512	200	0.23	0.18
	512	1,000	0.17	0.13
	512	6,325	0.16	0.11
	1,024	6,325	0.13	0.09

SSM	32 (95%)	50	0.58	-
	94 (95%)	200	0.42	-
	180 (95%)	1,000	0.30	-
	269 (95%)	6,325	0.24	-
	1298 (99%)	6,325	0.13	-

There are no average symmetric surface distance(ASSD) results for cartilage reconstruction using the statistical shape model (SSM) because the SSM does not create a cartilage surface. SSM results are for the number of principal components needed to explain 95 and 99% of the variance. NSM: neural shape model.

**Fig. A1.**
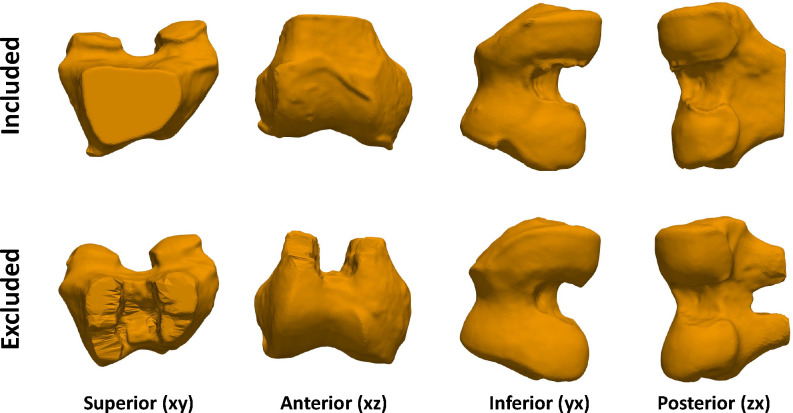
Visualization of the four images created and reviewed for all bones. The top row shows an example of a bone that was deemed fine and included. The bottom row was a bone that was excluded because of a hole in the middle of the bone - the location and size of the hole indicates the errors could be from screws in the knee used during anterior cruciate ligament reconstruction.

**Fig. A2.**
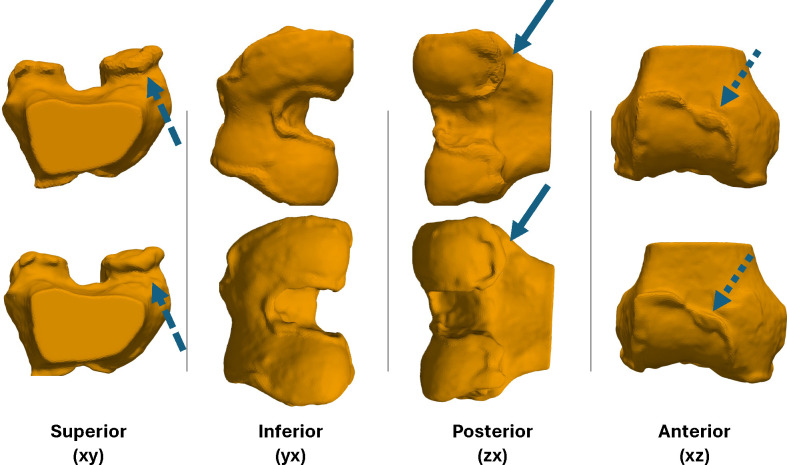
Example of artifacts introduced during nonrigid registration. The bottom row is the downsampled mesh (20,000 vertices) and the top row is the same surface after the template was non-rigidly registered to it. The blue arrows point to areas of coarsened triangles introduced because these diseased regions (osteophytes) do not exist in the template. This coarsening indicates that the registration is uniquely identifying that this small region is not present in the template and is likely beneficial in highlighting areas of high variance and thus might enhance disease prediction tasks. However, this likely reduces the capacity to reconstruct these regions accurately. These artifacts can occur to varying degrees depending on the severity of the osteophyte deformity.

**Fig. A3.**
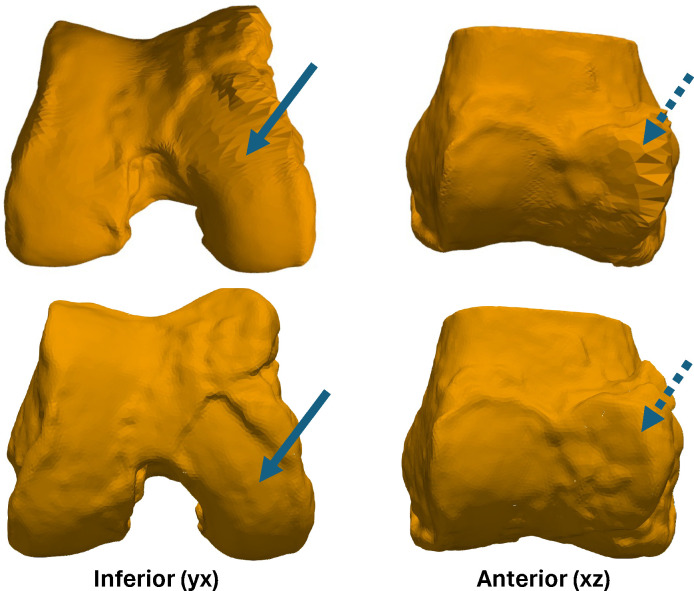
The bottom row is the downsampled mesh (20,000 vertices) and the top row is the same surface after the template was non-rigidly registered to it. The blue arrows point to areas of error or artifact. The errors on the left with the solid arrow highlight a region that almost always is accurately depicted in registration. However, in this one instance there is a small depression in the bone in that region that is abnormal; it is likely this depressed region that caused errors in finding appropriate correspondence and thus produced a poor match. The dashed arrows on the right show regions of coarsened triangles that are more likely an artefact than an explicit error, similar to Fig. A2

**Fig. A4.**
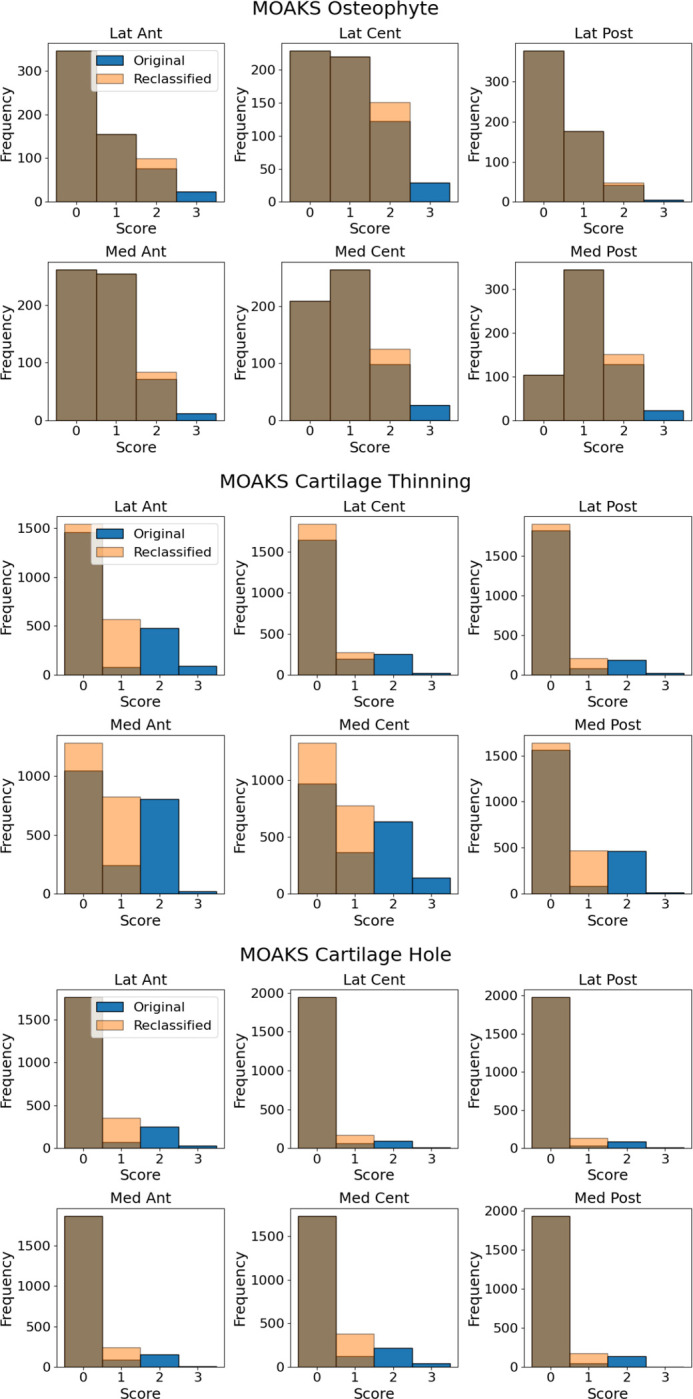
Histograms for the MRI Osteoarthritis Knee Score (MOAKS) before (blue) and after (orange) reclassification to handle imbalanced data. Each score started with 4 classes, the osteophytes score was classified to 3 levels (0–2) combining levels (2/3). The two cartilage scores were binarized; cartilage thinning was classified into < 10% thinning (level 0/1) and ≥ 10% thinning (level 2/3), and cartilage hole was binarized into no hole (level 0) and any hole (> 0). Lat: lateral, Med: medial, Ant: anterior, Cent: central, Post: posterior.

**Fig. A5.**
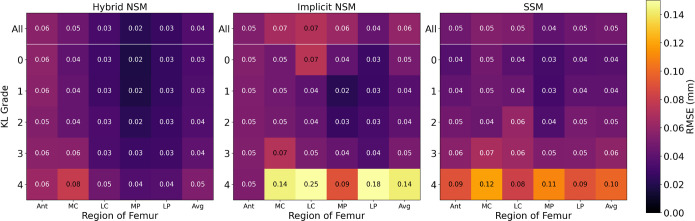
Visualization of the root mean squared error (RMSE) for cartilage thickness of each region computed between the reconstructed mesh and the reference mesh. Rows of results are presented all of the testing data (All) and for all subjects in each Kellgren Lawrence (KL) grade. Ant: anterior, MC: medial central, LC: lateral central, MP: medial posterior, LP: lateral posterior, Avg: average.

**Fig. A6.**
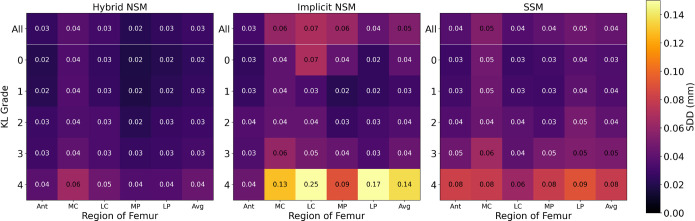
Visualization of the standard deviation of the difference (SDD) for cartilage thickness of each region computed between the reconstructed mesh and the reference mesh. SDD is a measure of consistency between the two measurements (reference, reconstructed) and is not influenced by bias. There are rows of results for all of the testing data (All) and for all subjects in each Kellgren Lawrence (KL) grade. Ant: anterior, MC: medial central, LC: lateral central, MP: medial posterior, LP: lateral posterior, Avg: average.

**Fig. A7.**
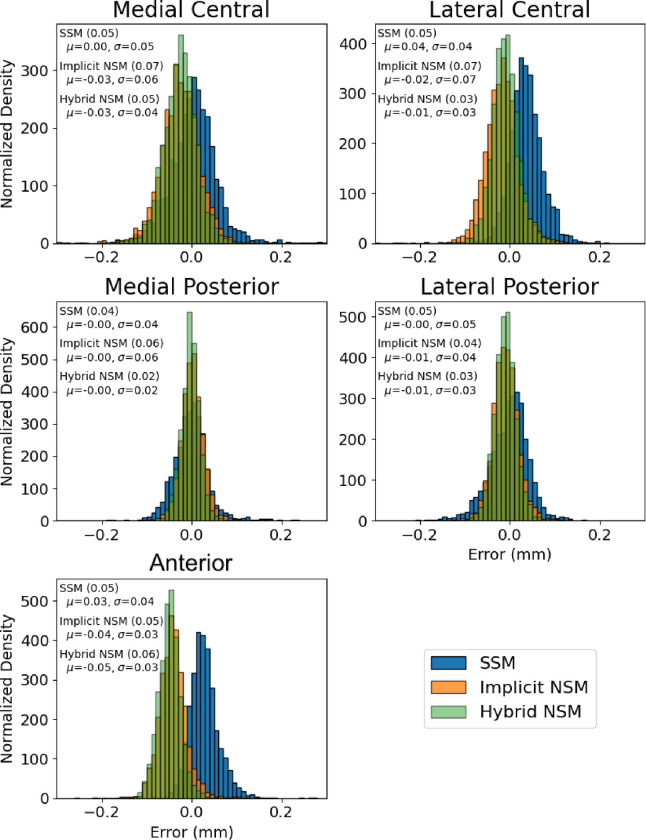
Histograms of the reconstruction errors for each model and region. Each plot is annotated with the root mean squared error in brackets after the model name, as well as the mean μ and standard deviation σ of that models’ errors. Generally, the NSMs had biases in the same direction, and the SSM biases were in the opposite direction.

**Fig. A8.**
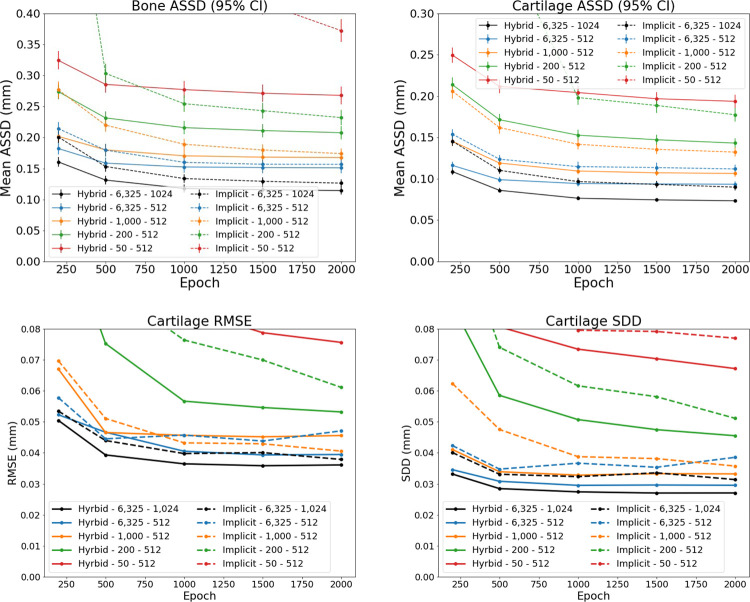
Reconstruction performance determined on the validation set (n=141). Legends indicate the model type (hybrid/implicit NSM, dataset size, and latent size). Solid lines represent the hybrid NSM, and dashed lines the implicit NSM. Error bars are 95% confidence intervals (CI) on the mean; non-overlapping error bars indicate statistical significance at p=0.05. Separate plots are created for bone and cartilage surface average symmetric surface distance (ASSD), and for the average cartilage biomarker root mean squared error (RMSE) and standard deviation of the difference (SDD).

**Fig. A9.**
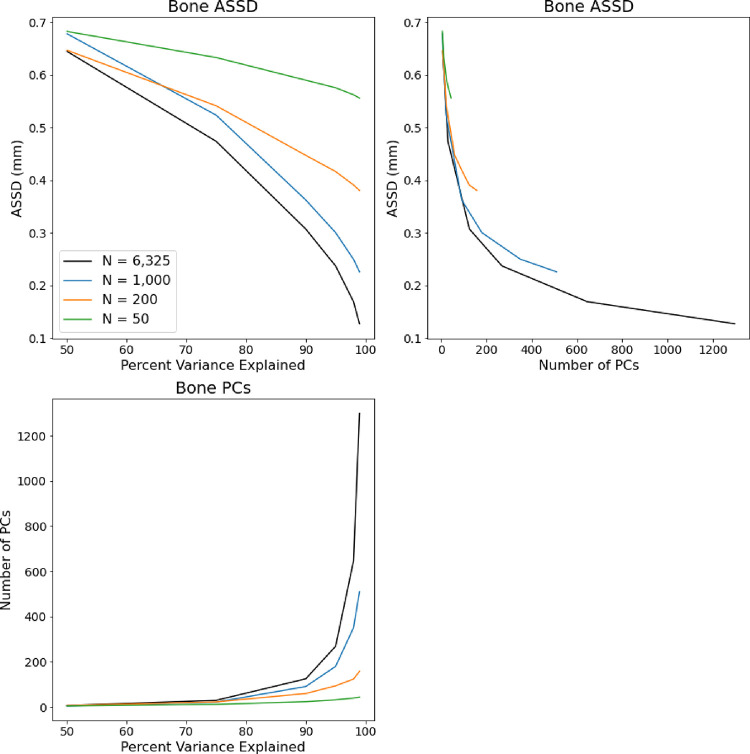
Visualization of the effect of increasing PC latent space size (number of principal components) on bone reconstruction accuracies measured using average symmetric surface distance (ASSD). The top plots use different x-axis with the right one showing the number of principal components (PC) and the left one showing the number of PCs as percent of explained variance. The bottom plot shows the relationship between percent explained variance and the number of PCs.

**Fig. A10.**
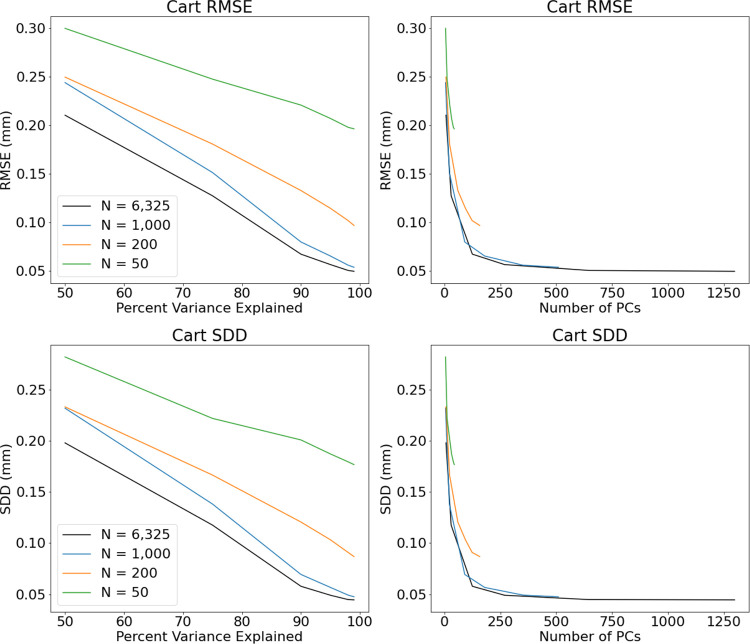
Visualization of the cartilage biomarker errors averaged over regions measured using root mean squared error (RMSE) and standard deviation of the difference (SDD). The top row is the RMSE results, the bottom row is the SDD results. The left column uses percent explained variance as the x-axis and the right column uses the number of principal components (PC) as the x-axis. When the percent explained variance is used as the x-axis, the biomarker errors linearly improve, and when number of PCs is used the performance exponentially decays.

**Fig. A11.**
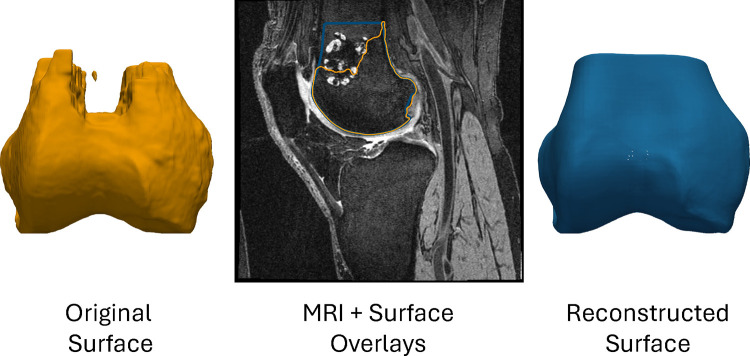
The left image shows the original surface reconstructed from the segmentation. The right image shows the reconstruction of the erroneous surface using the hybrid NSM; this reconstruction shows that it fills in the corrupted region. The middle image shows both surfaces overlaid on the original MRI data demonstrating that the NSM learned prior creates a plausible reconstruction for the missing portion of the femur bone.

**Fig. A12.**
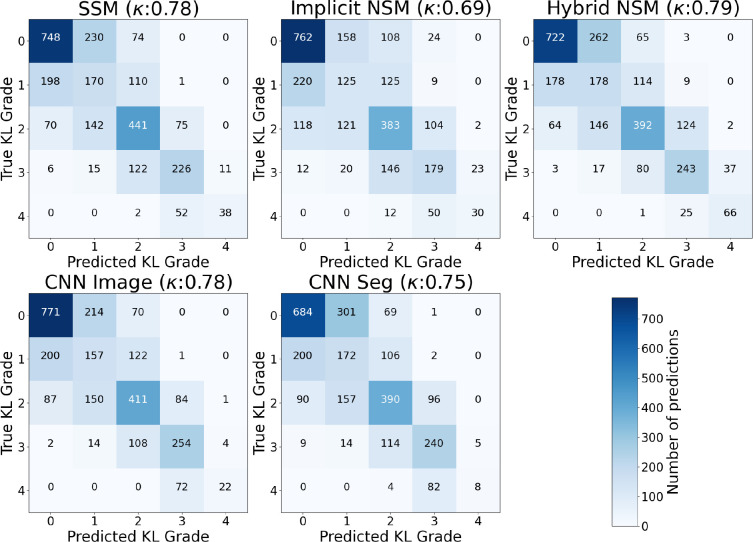
Confusion matrices of each model’s performance predicting Kellgren Lawrence (KL) osteoarthritis grade. Titles are annotated with the quadratic kappa *κ*. The implicit NSM performed worst.

**Fig. A13.**
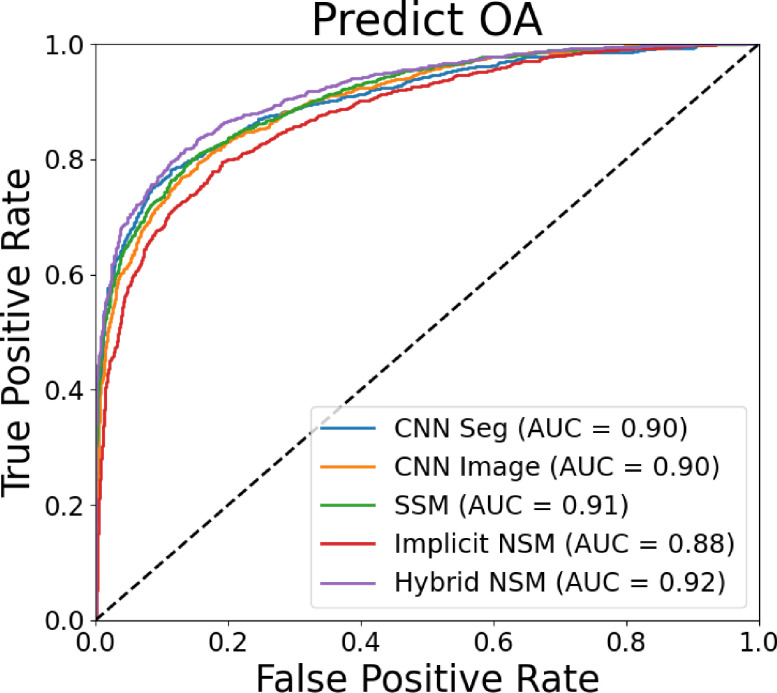
Visualization of the receiver operating characteristic curve for each model type. The hybrid neural shape model (NSM) performed best.

**Fig. A14.**
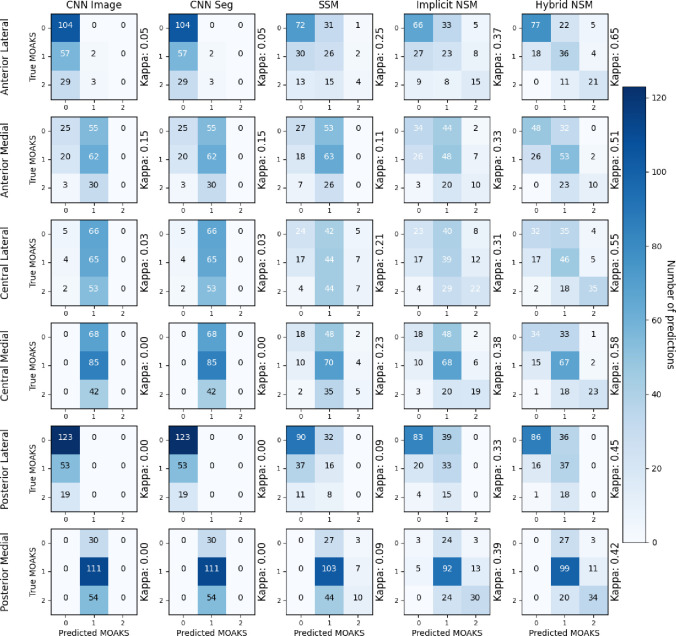
Confusion matrices displaying the performance of each model predicting osteophyte features (0=None, 1=small, 2=medium & large) in each of the 6 regions of interest. Columns are the different models, rows are the different regions. The right side of each plot is annotated with that model/region’s quadratic kappa *κ*

**Fig. A15.**
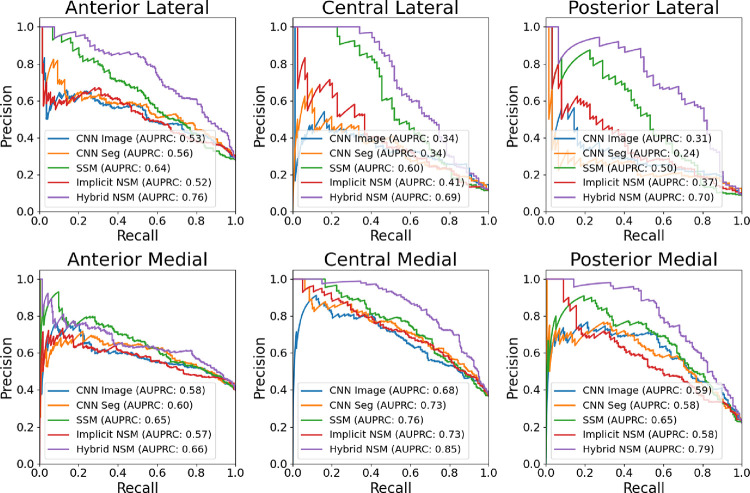
Visualization of performance predicting cartilage thinning for each of the six cartilage regions using precision-recall curves (AUPRC). The hybrid NSM performed best for every region. Models performed particularly well for the anterior lateral and central medial regions.

**Fig. A16.**
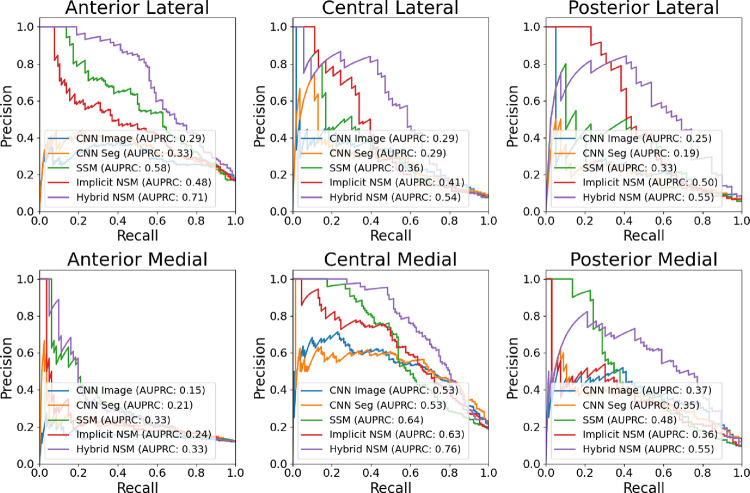
Visualization of performance predicting cartilage holes for each of the six cartilage regions using precision-recall curves. The hybrid NSM performed best for every region.

**Fig. A17.**
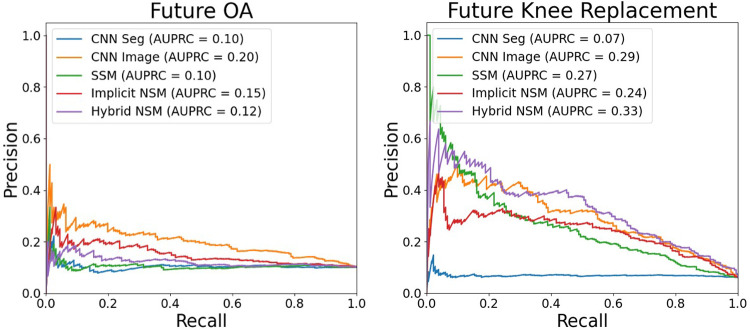
Precision recall curves show prediction performance for both future prediction tasks (OA diagnosis, knee replacement). None of the models did well for future OA prediction in terms of area under the precision-recall curve (AUPRC); all models did modestly for knee replacement prediction.

**Fig. A18.**
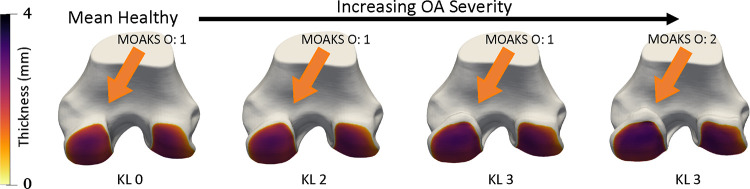
Interpolation in hybrid NSM shape space along the mean healthy to mean severe OA axis. Visualization is of the posterior (back) of the femur. The orange arrows point to an area of progressive osteophyte (O) growth and show the MOAKS O score specific to that region (posterior medial), which increases from grade 1 to grade 2 through the interpolation. The interpolation shows a smooth shape transformation across disease states and a concurrent increase in the severity of localized disease features. Each bone is labelled with the Kellgren Lawrence (KL) grade predicted by the logistic classifier.

**Fig. A19.**
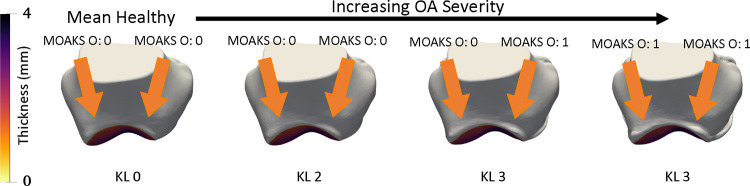
Interpolation in hybrid NSM shape space along the mean healthy to mean severe OA axis. Visualization is of the anterior (front) of the femur. The orange arrows point to areas of progressive osteophyte (O) growth and show the MOAKS O score specific to that region (left = anterior lateral, right = anterior medial), which increases from grade 0 to grade 1 for both regions by the end of the interpolation. The interpolation shows a smooth shape transformation across disease states and a concurrent increase in the severity of localized disease features. Each bone is labelled with the Kellgren Lawrence (KL) grade predicted by the logistic classifier.

## APPENDIX

### A. Mesh Processing

Surface meshes were created for each subject using established methodologies and open source tools [46], [74], [75], [76] outlined in the following. First, binary segmentation masks for each tissue were Gaussian filtered (σ: bone=0.5mm, cartilage=0.1mm), and surfaces were extracted using a continuous marching contours algorithm at a threshold of 0.5 [77]; marching cubes was applied using the VTK implementation [74] via the pyMSKT library [46]. To ensure meshes were watertight, the Robust Watertight Manifold Surface Generation method [78] implemented in Point Cloud Utils [76] was used with a mesh resolution of 200,000. The watertight meshes were slightly dilated compared to the original mesh, thus each point of the manifold mesh was projected back onto the original surface to preserve the original topology. Once watertight meshes were created, they were decimated to have 50% of the vertices, resulting in ~ 250,000 vertices per bone mesh, and ~ 150,000 vertices per cartilage mesh, again using the Point Cloud Utils library [76]. These methods have been commonly used for analyses of bone and cartilage surfaces in osteoarthritis (OA) [35], [51]. Segmentations, generated surface meshes, and all code for generating the surfaces from the segmentations will be publicly shared.

### B. Cartilage Biomarker Calculation

Cartilage biomarker generation was done using standard definitions of anatomic regions of interest established in the literature [79], [48]. Thickness calculations were performed using a normal vector method that is well established in the literature and is comparable to other approaches (e.g., field lines, nearest neighbour) [80]. Processing was done in three steps:

1. **Divide cartilage into subregions.** Cartilage is divided into 5 subregions (Anterior, medial and lateral central, and medial and lateral posterior) [79], [48]. See Fig. 2 for visualization of the 5 cartilage regions. To divide the cartilage, we identified three anatomic points along image axes: anterior-posterior (forward-backward) x, inferior-superior (up-down) y, medial-lateral (side-to-side) z. The three points are:
  - The trochlear notch point along the x axis $x_{t}$ was determined by flattening the segmentation along the y-axis, flood filling, and then performing an iterative search for the most anterior (negative x) point of the posterior border of the cartilage between the medial and lateral femoral condyles.
  - The most posterior (backward; positive x axis) bone point in the x axis $x_{pb}$ is the femoral bone voxel with the most positive position.
  - The center of the medial and lateral tibial cartilage*c* along the z axis $z_{c}$.

These three points were then used to divide femoral cartilage into 5 subregions, creating a femoral cartilage subregion mask. i) Anterior cartilage is any point where $x<x_{t}$, ii) medial weight-bearing cartilage is any point where $x_{t}<x<x_{pb}\wedge z>z_{c}$, iii) lateral weight-bearing cartilage is any point where $x_{t}<x<x_{pb}\wedge z<z_{c}$, iv) medial posterior cartilage is any point where $x>x_{pb}\wedge z>z_{c}$, and v) lateral posterior cartilage is any point where $x>x_{pb}\wedge z<z_{c}$.. [80], [79], [48]

1. **Compute vertex-wise cartilage thickness.** Cartilage thickness was assigned to each bone vertex by projecting a vector normal to the surface and if the vector intersected the cartilage mesh twice, then the Euclidean norm of the intersections was calculated as the cartilage thickness and assigned to the respective bone vertex. Otherwise, a thickness of 0 was assigned.
2. **Assign vertex subregions.** Bone vertices with nonzero cartilage thickness values were assigned a cartilage region label. Vectors were again projected normal to the surface and the 3D cartilage subregion mask was probed 100 times along this vector between the two cartilage intersections. The bone vertex was assigned the label with the most frequent cartilage subregion determined from the probe.

### C. Mesh Quality Control

We generated static images from 4 orthogonal views (top/superior, bottom/inferior, front/anterior, back/posterior) of the registered versions of every bone mesh ( Sec. III-A.1 *Bone Surface Registration*) in the dataset and manually reviewed them. This process identified 57 bones with obvious and large errors, Fig. A1 includes examples of the static images used for quality control as well as of an excluded example.

#### 1) Registration artifacts.:

These quality control static images highlight errors or artifacts that can occur as a result of nonrigid registration. While the original downsampled meshes (20,000 vertices) used for shape modeling are normally clean with regularly spaced triangles, non-rigid registration can introduce artifacts when there is discordant anatomy between two surfaces. For example, Fig. A2 shows an example of a mesh with low-resolution on the mesh surface at diseased regions of the bone surface, this is likely at least in part to explain why the SSM-based approaches do not perform as well for reconstruction compared to the NSM-based approach. Another example Fig. A3 shows abnormal surface triangles in a generally smooth surface region, this was a rare artifact but highlights how registration-based approaches can fail leading to sub optimal results.

### D. Prediction Tasks Generation

During creation of the dataset, MRI Osteoarthritis Knee Score (MOAKS) grades were converted from 4 labels to 3 (osteophyte) or 2 (cartilage thinning, cartilage hole) labels due to data imbalances. Fig. A4 shows histograms of the MOAKS scores (osteophyte, cartilage thinning, cartilage hole) before and after reclassification.

The benchmark models used in this study included three shape models (implicit neural shape model (NSM), hybrid NSM, and a statistical shape model (SSM)) as well as two convolutional neural network (CNN) based approaches. Additional implementation and training details for each are provided below.

### E. Neural Shape Model

The NSM model was built using PyTorch. Training and inference were performed on a single graphics processing unit, either a Nvidia A6000 or 2080ti.

#### a) Training.:

During training, we used separate learning rates and schedules for the network weights and the latents z. For both sets of parameters, learning rate (lr) was decayed as $lr=lr_{0}\times f^{\left(e/i\right)}$ where $lr_{0}$ is the lr at time zero, f is the update factor e is the current epoch, and i is the interval which lr is updated. Network weights had parameters of $lr=5\times 10^{-3}$, f=(1/1.05), i=16.67 and latents z had parameters of $lr=10^{-4}$, f=0.1, i=1000.

The latent regularization weight from Eq. (1) was ($1/\sigma^{2}=10^{-4}$; σ=100. The weight had a linear warmup over the first 100 epochs and was then cyclically annealed with 5 cycles over the training period (2,000 epochs). The cyclic anneal weight β for each cycle was defined using Eq. (A1) where t is the epoch for the current cycle and T is the number of epochs in each cycle (2000*/*5). We clamped signed distances *s* at |s|=0.1 for the implicit NSM and |s|=1 for the hybrid NSM.

(A1)
$$\beta (t)=\left\{\begin{array}{ll} 2\frac{t}{T} & \text{if}\;0\leq t<\frac{T}{2}\\ 1 & \text{if}\;\frac{T}{2}\leq t<T \end{array}\right.$$

#### b) Reconstruction.:

First, the bone/cartilage pair to be reconstructed were similarity registered to the mean bone shape of the NSM and then the bone/cartilage surfaces were scaled to be within a unit sphere, the same as during training Sec. IV-A. Then, the network weights were frozen and a randomly initialized latent z was optimized to reconstruct the shape. Specifically, for each iteration, 20,000 points were randomly sampled from the surface, and an ${ℒ}_{1}$ loss between the network predicted signed distance s and the actual distance (0) was optimized using the Adam optimizer. The lr was decayed by a factor of 0.9 every 20 epochs. Optimization was performed for a maximum of 2,000 epochs with a patience of 50 epochs. Predicted *s* were clamped at |s|=0.1. No latent regularization was used.

#### c) Hybrid NSM:

The hybrid NSM is similar to previous work [25], [26]. The overall architecture is described in Fig. 4. A global latent z was passed through a multilayer perceptron (MLP) yielding a vector of length 2048 that was reshaped to 2 × 2 × 512 using a fully connected layer and input into a CNN. Our CNN had 5 2D transpose convolution layers, with stride 2 and 512 channels as outputs. The final output layer of the CNN was sized 64 × 64 × 384; features maps were split into 128 per plane. For a given sampled point, a length 128 z was obtained per plane via bilinear interpolation and the zs were combined via summation. The local z and the sampled points xyz position were concatenated and input into a 3-layer MLP with width 512, *ReLU* activations, and a length two output with a tanh activation.

### F. Statistical Shape Model

Registered meshes described in paragraph III-A.1.b were used to fit the model using the available training data by creating a M×N matrix where M is the number of training examples and N is the number of columns/features. In our case, $N=\left(3+1\right)\times 20,\;000=80,000$ where 20,000 is the number of vertices, 3 represents XYZ dimensions, and 1 is for the cartilage thickness features stored at each vertex. Principal component analysis (PCA) was applied to this matrix to obtain eigenvectors v and eigenvalues λ. Normalized principal component (PC) scores for training and testing data were obtained by projecting registered points onto v and normalizing by $\sqrt{\lambda }$. Bone surfaces, and coinciding cartilage thickness per vertex, were reconstructed by $recon=\sum_{i=1}^{k}\;\left({PC}_{i}\times \sqrt{\lambda_{i}}\right)\cdot v$ where k is the number of PCs used in the reconstruction.

### G. Convolutional Neural Network

We trained a 3D DenseNet121 CNN on two distinct types of medical imaging data: raw DICOM images and segmentation masks. This approach allowed us to evaluate the significance of pixel magnitude information.

We used an input size of 384 × 384 × 80 and output channels being equal to the product of the number of tasks and classes per task. To accommodate our requisite prediction tasks, six model variants were devised one for each of the following tasks: OA Staging, OA Diagnosis, Future OA Incidence, Future knee replacement, MOAKS Ostephytes, and finally MOAKS Cartilage Thinning and MOAKS Cartilage Hole as a single model.

Image pre-processing included normalization of each individual image to have a mean of 0 and a standard deviation of 1 as well as standardization of image orientation. so that the first dimension extends from left to right, the second from posterior to anterior, and the third from inferior to superior. Inputs were then resampled to a uniform spacing of 0.3645 mm in-plane and 1.4 mm out-of-plane using bilinear interpolation. Subsequently, the images were center-cropped and padded to dimensions of 384 × 384 in-plane and 80 out-of-plane.

To optimize the CNNs, we used the AdamW optimizer, with initial $lr=10^{-5}$, exponential decay with gamma=0.8, and weight decay of 0. Training used the same loss functions as the shape-model MLPs Tab. A1 and a batch size of 8. Trainnig was performed on a single Nvidia A6000 GPU.

### H. Reconstruction

#### a) Cartilage Biomarkers:

Cartilage biomarkers computed from reconstructions were compared to the original mesh calculations. Absolute error was determined using root mean squared error (RMSE) for all regions, and separately for each KL grade Fig. A5. Similarly, the standard deviation of the difference (SDD) was calculated by disease category and regions Fig. A6; the SDD provides a measure of consistency that accounts for bias between the methods. Consistently better SDD compared to RMSE indicates that each method had a small bias compared to the reference segmentation method, this is confirmed by visualizing the distributions of errors between the ground truth and the reconstructed surfaces Fig. A7. It is also apparent that different regions and models had different biases. For example, in the anterior femur, the NSMs underestimated thickness, whereas the SSM overestimated thickness.

During training, larger datasets, larger latent sizes, and hybrid (vs. implicit) architectures improved reconstructions. Specifically, bone and cartilage surface reconstruction accuracies were measured using ASSD, and cartilage biomarker reconstructions were compared to the reference using RMSE and SDD Fig. A8. These figures showed that increasing dataset size increased performance. Increasing the latent size (black lines) considerably improved reconstruction performance.

Reconstruction with the SSM using progressively more PCs improved reconstruction of surface ASSD ( Fig. A9), and cartilage biomarkers ( Fig. A10) up to the the number of components needed to explain 99% of the variance in the original dataset. When plotted with the x-axis as the percent explained variance, it appears as though ASSD is exponentially improving even at 99% explained variance. However, the number of PCs required for a given increase in explained variance is increasing exponentially. When ASSD is plotted as function of the number of PCs it is apparent that ASSD improvement is actually exponentially decaying Fig. A9.

#### b) Reconstructing Erroneus Surfaces.:

To determine how a trained NSM reconstructs a mesh with artifacts, we used the fitted hybrid NSM to reconstruct the surface of one of the 57 meshes excluded due to obvious errors. Fig. A11 shows surfaces of the original erroneous surface, the reconstructed surface, and an overlay of both surfaces on the original MRI. These data demonstrate that the NSM faithfully reconstructs plausible anatomical surfaces while filling in the corrupted regions with reconstructions based on the learned priors. This finding supports previous research which indicates that NSMs can be used as a means of refining automated segmentations to ensure anatomic plausibility [38].

### I. Prediction Tasks

MLP hyperparameters were determined via a grid search over depth (2,3), width (64–256), dropout (0.2, 0.4), lr (10^−3^ to 10^−5^), and batchsize (64–512). The two future prediction tasks (knee replacement, osteoarthritis) were trained with a class imbalance weight. MLP hyperparameters were determined separately for each task, but the same parameters were used across shape models (SSM, hybrid NSM and implicit NSM). Specific parameters are described in Tab. A1. Different tasks leveraged different loss functions. OA diagnosis, MOAKS cartilage thinning, and MOAKS cartilage hole all used binary cross entropy. Future knee replacement (KR) and future OA diagnosis both used binary cross entropy with the positive class (smallest) weighted inversely proportional to its relative occurrence. KL grading and MOAKS osteophyte scores both used an ordinal regression loss [63].

#### a) Disease Staging.:

To visualize differences in KL grading between the methods, we plot confusion matrices for each of the shape and the CNN models Fig. A12. All models had good performance with quadratic weighted kappa κ≥0.69.

#### b) Disease Diagnosis.:

All models performed well predicting OA with area under the receiver operating characteristic curve (AUROC) ≥ 0.88 Fig. A13.

#### c) Advanced Disease Diagnosis.:

MOAKS scores per region are provided in Figs. A14 to A16. The hybrid NSM performed best for all three MOAKS prediction tasks. Cartilage predictions performed best for the anterior lateral and the central medial regions, which are also common locations of cartilage deterioration in OA Figs. A15 and A16.

#### d) Future Prediction.:

All models performed poorly for future OA disease prediction and performed better for future knee replacement prediction Fig. A17. The hybrid NSM performed best for future knee replacement risk, and the CNN-image performed best for future OA prediction. Overall knee deformity is a decision factor of whether a patient receives knee replacement; the hybrid NSM, which better captured and localized shape features of OA disease Figs. A14 and A16 may have also better represented features common in individuals who undergo knee replacement. Better future disease prediction by the CNN on raw image data may be explained by it leveraging features other than shape, such as bone bruises or general joint inflammation Fig. A17.

### J. Interpretability

Interpolation in latent space from the mean healthy (KL 0) to the mean severe OA (KL 4) knees showed progressive increases in disease-specific features of cartilage Fig. 6, as well as osteophytes Figs. A18 and A19. Not only did the bones show a progressive increase in osteophyte sizes, but when the logistic classifiers were used they also predicted progressively higher osteophyte scores Figs. A18 and A19. As shown in all of the interpolation figures Figs. 6, A18 and A19, the first bone is correctly classified as KL = 0, however, the last bone is misclassified as KL = 3. Osteoarthritis is a heterogeneous disease with many different presentations leading to the same severity score (e.g., KL 4). It is likely that in this example the average of all subjects with KL 4 reduced extremes of any one presentation of OA thus resulting in moderate severity in most features and leading to an overall shape that was classified as KL 3 instead of KL 4.

**TABLE A1:** Hyperparameters used to train the multilayer perceptrons (MLP) that predicted each clinical outcome.

	Layers	Width	Dropout	LR	Batchsize	Loss
	
OA	2	256	0.4	10^−5^	128	BCE
KL	2	256	0.4	10^−4^	128	CORN
MOAKS Osteophyes	3	256	0.2	10^−3^	512	CORN
MOAKS Cart Thinning	3	128	0.4	10^−3^	512	BCE
MOAKS Hole	2	256	0.4	10^−4^	256	BCE
Future OA	2	256	0.4	10^−5^	128	wBCE
Future KR	2	256	0.2	10^−5^	64	wBCE

BCE: binary cross entropy, wBCE: weighted BCE, LR: learning rate, OA: osteoarthritis, KL: Kellgren Lawrence, Moaks: MRI osteoarthritis knee score, KR: knee replacement, CORN: Consistent Rank Logits ordinal regression loss
